# Transcriptional perturbation of protein arginine methyltransferase-5 exhibits MTAP-selective oncosuppression

**DOI:** 10.1038/s41598-021-86834-7

**Published:** 2021-04-01

**Authors:** Sara Busacca, Qi Zhang, Annabel Sharkey, Alan G. Dawson, David A. Moore, David A. Waller, Apostolos Nakas, Carolyn Jones, Kelvin Cain, Jin-li Luo, Adriana Salcedo, Iris Chiara Salaroglio, Chiara Riganti, John Le Quesne, Tom John, Paul C. Boutros, Shu-Dong Zhang, Dean A. Fennell

**Affiliations:** 1 Leicester Cancer Research Centre, University of Leicester, Leicester, UK; 2 Chongqing Key Laboratory of Translational Research for Cancer Metastasis and Individualized Treatment, Chongqing University Cancer Hospital and Chongqing Cancer Institute and Chongqing Cancer Hospital, Chongqing, China; 3 Cancer Research UK Lung Cancer Centre of Excellence, University College London Cancer Institute, London, UK; 4 Department of Cellular Pathology, University College London Hospital, London, UK; 5 Department of Thoracic Surgery, St. Bartholomew’s Hospital, London, UK; 6 Department of Thoracic Surgery, Glenfield Hospital, University Hospitals of Leicester, Leicester, UK; 7 MRC Toxicology Unit, University of Cambridge, Leicester, UK; 8 Departments of Human Genetics and Urology, Jonsson Comprehensive Cancer Center and Institute for Precision Health, University of California, Los Angeles, USA; 9 Ontario Institute for Cancer Research, Toronto, Canada; 10 Departments of Medical Biophysics and Pharmacology and Toxicology, University of Toronto, Toronto, Canada; 11 Department of Oncology, University of Torino, Torino, Italy; 12 Peter MacCallum Cancer Centre, Melbourne, Australia; 13 Northern Ireland Centre for Stratified Medicine, School of Biomedical Sciences, Ulster University, Londonderry, UK

**Keywords:** Mesothelioma, Cancer, Targeted therapies, Cell biology, Cell growth

## Abstract

We hypothesized that small molecule transcriptional perturbation could be harnessed to target a cellular dependency involving protein arginine methyltransferase 5 (PRMT5) in the context of methylthioadenosine phosphorylase (MTAP) deletion, seen frequently in malignant pleural mesothelioma (MPM). Here we show, that MTAP deletion is negatively prognostic in MPM. In vitro, the off-patent antibiotic Quinacrine efficiently suppressed PRMT5 transcription, causing chromatin remodelling with reduced global histone H4 symmetrical demethylation. Quinacrine phenocopied PRMT5 RNA interference and small molecule PRMT5 inhibition, reducing clonogenicity in an MTAP-dependent manner. This activity required a functional PRMT5 methyltransferase as MTAP negative cells were rescued by exogenous wild type PRMT5, but not a PRMT5E444Q methyltransferase-dead mutant. We identified c-jun as an essential PRMT5 transcription factor and a probable target for Quinacrine. Our results therefore suggest that small molecule-based transcriptional perturbation of PRMT5 can leverage a mutation-selective vulnerability, that is therapeutically tractable, and has relevance to 9p21 deleted cancers including MPM.

## Introduction

Deletion of chromosome 9p21 encompasses the tumour suppressors CDKN2A and methylthioadenosine phosphorylase (MTAP)^1,2^, and is a frequent somatic event in several cancers^1,2^. MTAP is a critical enzyme in the methionine salvage pathway that metabolizes the substrate methylthioadenosine (MTA), leading to regeneration of methionine and adenosine^3^. Deletion of MTAP has been reported to confer a vulnerability to inhibition of the epigenetic regulator protein arginine methyltransferase 5 (PRMT5), which symmetrically methylates arginine on histone H4, leading to chromatin remodelling^2,4,5^.

Malignant pleural mesothelioma (MPM) is an incurable cancer caused by asbestos that lacks effective targeted therapies, and harbours high frequency deletion of 9p21^6,7^. Here we report a negative prognostic impact of MTAP on survival of patients with MPM, and demonstrate potential to selectively target somatic deletion of MTAP using a small-molecule based strategy, involving PRMT5 transcriptional suppression.

## Results

### MTAP is negatively prognostic in MPM

**Figure 1:**
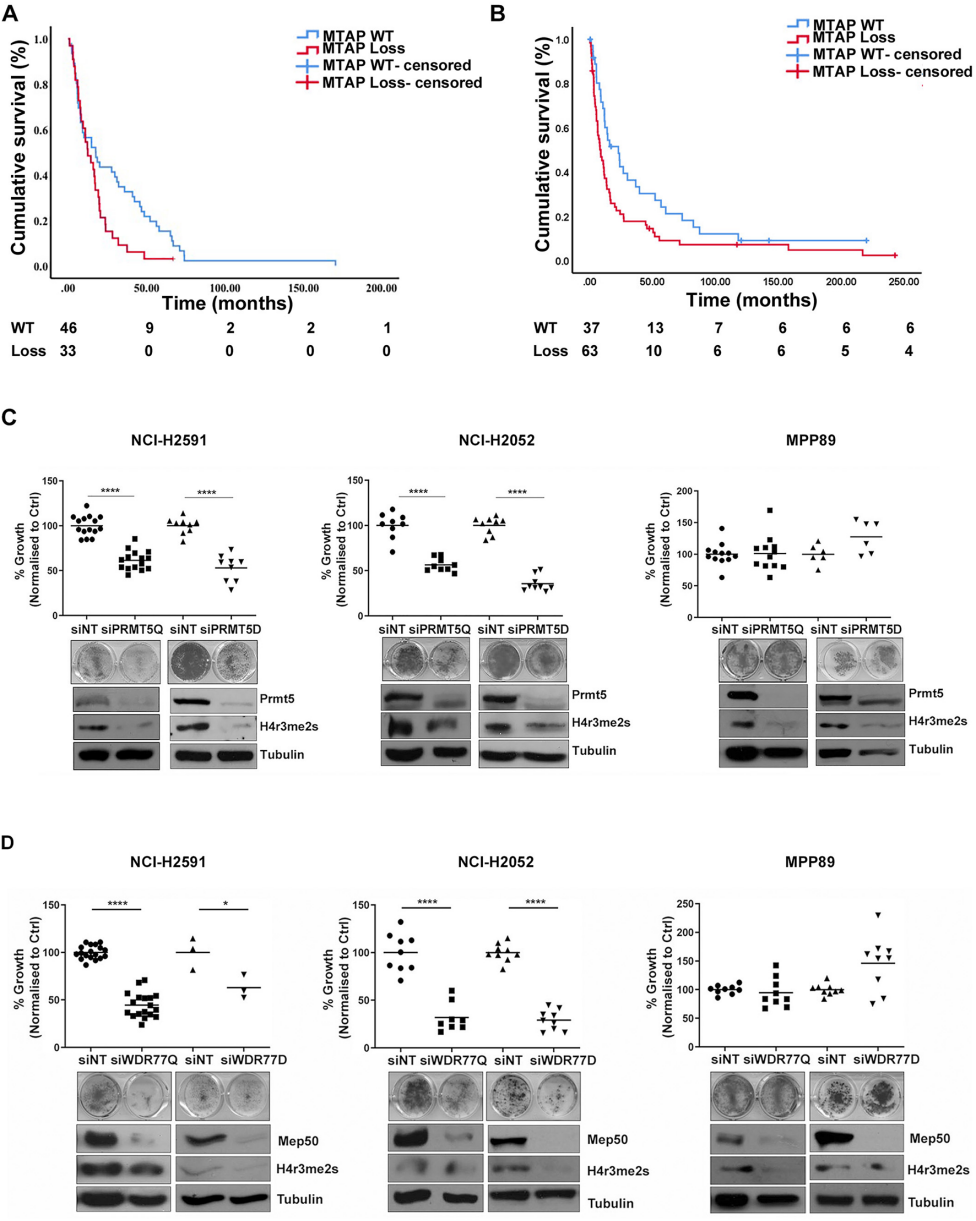
MTAP is negatively prognostic in MPM (**A**) Kaplan–Meier survival curve for OS of MTAP positive and MTAP negative patients (n = 79). (**B**) Kaplan–Meier survival curve for OS of MTAP positive and MTAP negative patients from validation cohort (n = 100). (**C**) MTAP negative cells (NCI-H2052, NCI-H2591) and MTAP positive cells (MPP89) were transfected with siNT or siPRMT5 20 nM (Q: Qiagen sequence, D: Dharmacon sequence). Cell proliferation was measured by clonogenic assay 5–7 days after transfection. Data were normalized to siNT controls (NCI-H2591: Q *p* = 0.0001 D *p* = 0.0001; NCI-H2052: Q *p* = 0.0001 D *p* = 0.0001; NCI-H2452: Q *p* = 0.0001 D *p* = 0.0001; MPP89: Q *p* = n.s. D *p* = n.s.). The levels of PRMT5 expression and H4 arginine 3 symmetrical di-methylation (H4R3me2S) were measured by western blot 72 h after transfection. These gels have been cropped and full length gels are presented in Supplementary Fig. 3A–C. **(D**) Cells were transfected with siNT or siWDR77 20 nM. Cell proliferation was measured by clonogenic assay 5–7 days after transfection. Data were normalized to siNT controls (NCI-H2591: *p* = 0.0001; NCI-H2052: *p* = 0.0001; NCI-H2452: *p* = n.s; MPP89: *p* = n.s.). The levels of PRMT5 expression and H4 arginine 3 symmetrical di-methylation (H4R3me2S) were measured by western blot 72 h after transfection. These gels have been cropped and full length gels are presented in Supplementary Fig. 3D–F.

MTAP copy number loss was assessed in 79 mesotheliomas acquired at radical surgery involving extended pleurectomy decortication (EPD) (Supplementary Table I). Copy number loss of 9p21.3 encompassing CDKN2A/MTAP was observed in 47% (37 samples) of patients and 42% had MTAP deletion (33 samples); co-deletion of MTAP and CDKN2A was frequent (95%). MTAP heterozygous or homozygous deletion was associated with shorter median overall survival (OS) compared to wild-type MTAP, 12.5 (95%CI 6.8–18.2) versus 17.6 (95%CI 6.5–28.7) months respectively, *p* = 0.042, HR 0.609 (95%CI 0.376–0.987), (Fig. 1A). In an independent validation cohort of 100 samples (Supplementary Table I), 63% of patients harboured MTAP deletion (63 samples); co-deletion of MTAP and CDKN2A was similarly at high frequency (83%). MTAP deletion was associated with shorter overall survival (OS) compared to wild-type MTAP, 8.7 (95%CI 4.6–12.8) versus 22.7 months (95% CI 11.4–33.9) months respectively, *p* = 0.021, HR 0.599 (95%CI 0.386–0.930) (Fig. 1B).

To explore MTAP as a covariate, we performed a univariate analysis showing a statistically significant effect between overall survival with: age (*p* = 0.02); iMig stage (*p* = 0.009) and MTAP status (*p* = 0.008). These variables were taken forward into a Cox multivariate analysis showing that iMig stage (HR 1.36 (95%CI 1.11–1.68), *p* = 0.003) and MTAP status (HR 1.41 (95%CI 1.03–1.93), *p* = 0.032) retained statistical significance whilst age (HR 1.32 (95%CI 0.97–1.79), *p* = 0.08) did not.

### PRMT5 silencing mediates growth arrest in MTAP negative mesothelioma

To determine whether MTAP negative MPM cells were dependent on PRMT5, we silenced PRMT5 expression by RNA interference in both MTAP wild-type and negative MPM cell lines (Fig. 1C). Reduced clonogenic growth was selective for MTAP negative cell lines, with concurrent reduction in symmetrical di-methylation of Histone H4 arginine 3 (H4R3me2S). Silencing of the PRMT5 interactor WDR77 phenocopied PRMT5 silencing in MTAP negative cells, leading to a reduced clonogenic activity and a reduced H4R3me2S (Fig. 1D). This effect was not phenocopied by siRNA targeting the PRMT5 interactor RIO1 kinase (Supplementary Fig. 1A).

**Figure 2:**
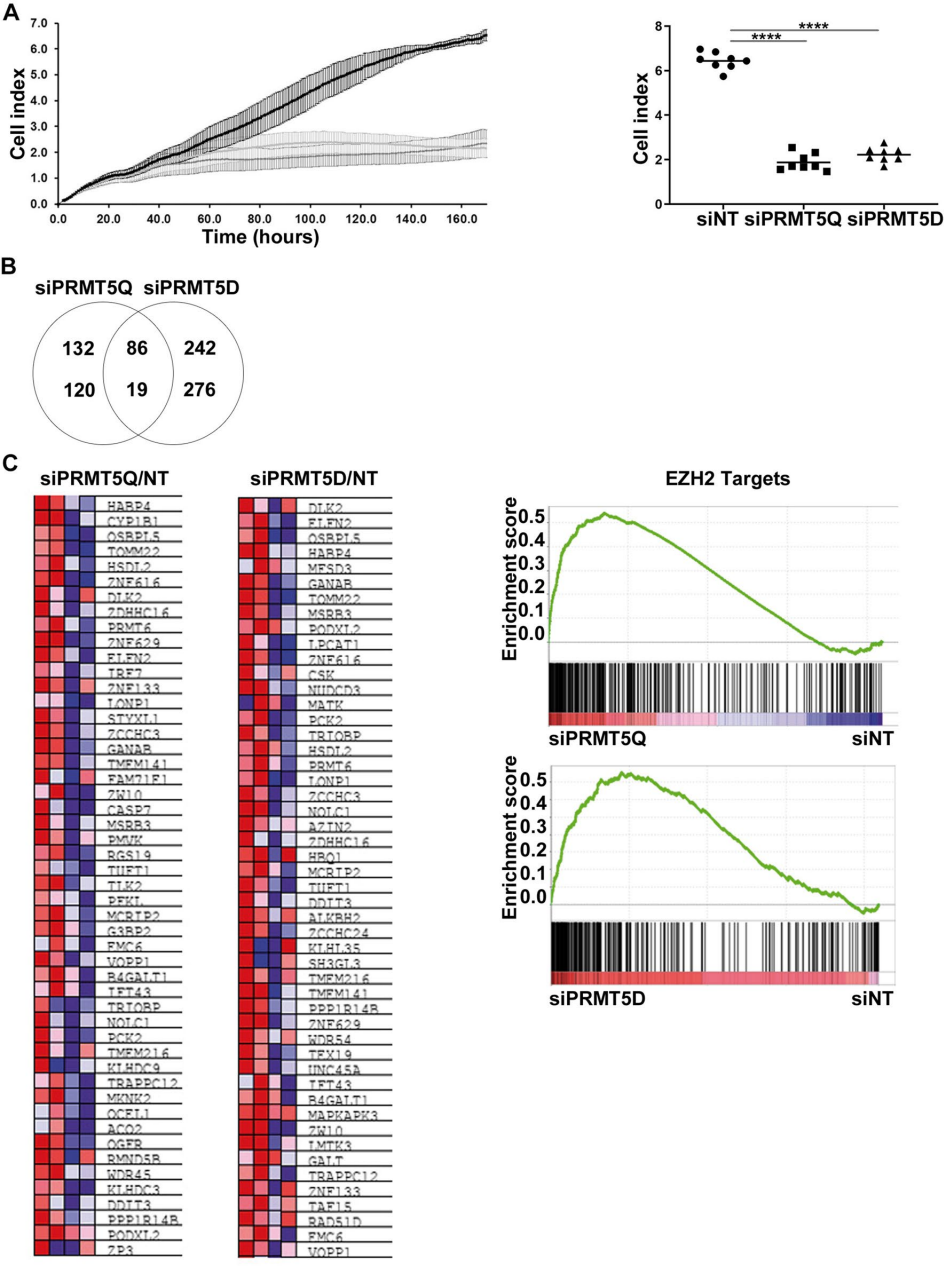
PRMT5 silencing mediates growth arrest in MTAP negative mesothelioma. (**A**) NCI-H2591 cells were transfected with siNT or siPRMT5 (Q: qiagen sequence, D: Dharmacon sequence) 20 nM. Cell proliferation was measured for 168 h with the excelligence real-time cell analyser. Data were normalized to siNT controls (Q *p* = 0.0015 D *p* = 0.0019). (**B**) Venn diagram showing upregulated and downregulated genes comparing the PRMT5 siRNAs versus siNT. (**C**) Heatmaps and representative GSEA plots showing a significantly enriched up regulated signatures (siPRMT5 vs. siNT).

To investigate the kinetics of growth arrest, we conducted real time analysis following PRMT5 RNAi which showed a growth arrest after 120 h (Fig. 2A). Neither apoptosis nor cell cycle perturbation were observed by flow cytometry after PRMT5 silencing (Supplementary Fig. 1B).

To determine whether or not PRMT5 silencing-induced chromatin remodelling would upregulate tumour suppressor pathways, we conducted gene expression, examining canonical pathways linked to upregulated genes. Overexpression of tumour suppressors, such as EIF3F, FOXP4, ZBTB4, GANAB, TMEM141 was observed. Gene set enrichment analysis revealed a significant enrichment of the EZH2 target gene (Fig. 2B,C, Supplementary Table 2).

In common with PRMT5 siRNA, the small molecule EPZ015666 reduced clonogenic growth (Supplementary Fig. 1C). Neither apoptosis nor cell cycle perturbation were observed by flow cytometry after inhibition by EPZ015666 (Supplementary Fig. 1D).

### Quinacrine hydrochloride is a PRMT5 perturbagen

**Figure 3:**
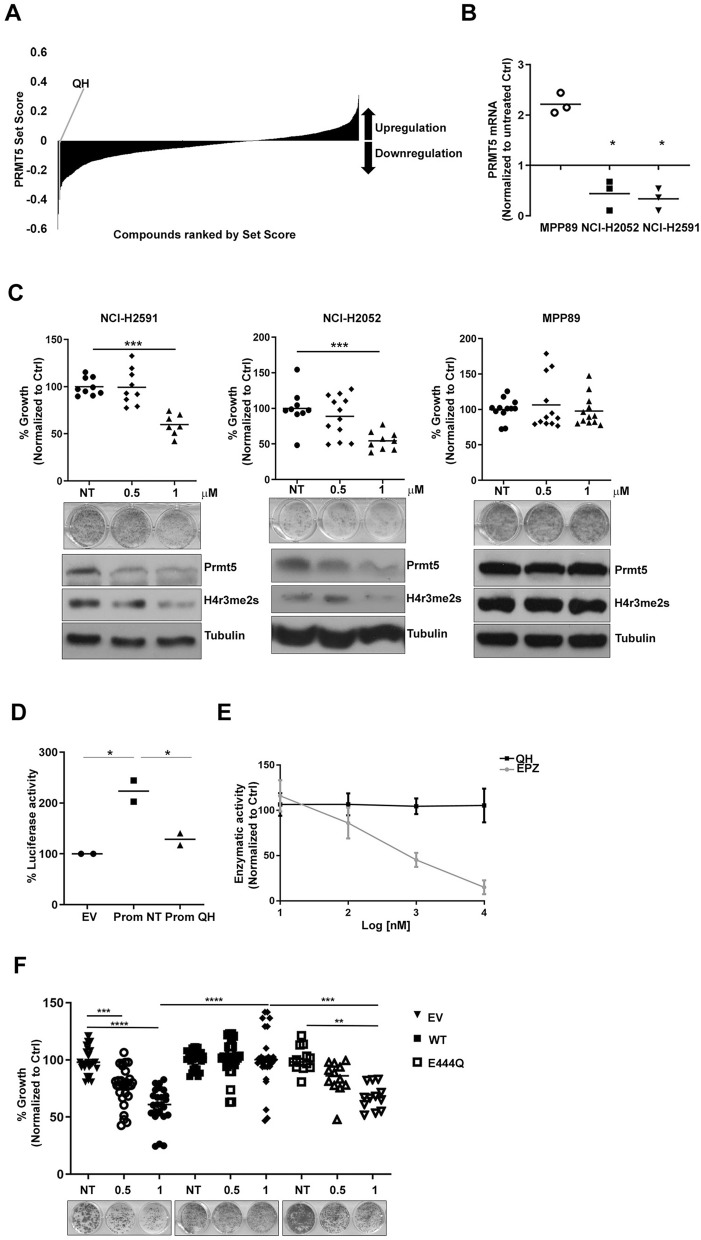
Identification of Quinacrine Hydrochloride as a PRMT5 perturbagen. (**A**) Connectivity mapping analysis showing quinacrine as transcriptional suppressor of PRMT5. (**B**) PRMT5 mRNA expression was evaluated by qRT-PCR on RNA extracted from cells treated with Quinacrine 1 µM for 72 h. Data were normalized to untreated control (NCI-H2052 *p* = 0.0308; NCI-H2591: *p* = 0.0063; MPP89 *p* = 0.005). (**C**) Cells were left untreated or treated with Quinacrine 0.5 µM and 1 µM for 72 h. Cell proliferation was measured by clonogenic assay 5–7 days after treatment. Data were normalized to untreated controls (NCI-H2591: 0.5 µM *p* = n.s. 1 µM *p* = 0.0001; NCI-H2052: .5 µM *p* = n.s. 1 µM *p* = 0.0017; MPP89: 0.5 µM *p* = n.s. 1 µM *p* = n.s.). The levels of PRMT5 expression and H4 arginine 3 symmetrical di-methylation (H4R3me2S) were measured by western blot 72 h after transfection. These gels have been cropped and full length gels are presented in Supplementary Fig. 3G–I. (**D**) The PRMT5 promoter activity was measured by a luciferase reporter assay in NCI-H2591 cells transfected with pGL2 basic (EV) or pGL2-PRMT5 and then treated with Quinacrine 1 µM for 72 h. Data were normalized to pGL2 basic (pGL2 basic vs. pGL2-PRMT5 NT *p* = 0.0162; pGL2 basic vs. pGL2-PRMT5 1 µM *p* = n.s.; pGL2-PRMT5 NT vs. pGL2-PRMT5 1 µM *p* = 0.0335). (**E**) Cells were left untreated or treated with Quinacrine or EPZ015666 10 nM, 100 nM, 1 µM and 10 µM. PRMT5 enzymatic activity was measured after 2 h. (**F**) NCI-H2591 cells were transfected with GFP empty vector, PRMT5 WT and PRMT5 E444Q and left untreated or treated with Quinacrine 0.5 µM and 1 µM for 72 h. Cell proliferation was measured by clonogenic assay 5–7 days after treatment. Data were normalized to untreated controls (Empty vector: NT vs. 0.5 µM *p* = 0.0004, NT vs1 µM *p* < 0.0001; PRMT5 E444Q: NT vs. 1 µM *p* = 0.0026; Empty vector 1 vs. PRMT5 WT *p* < 0.0001).

To fully harness the potential for MTAP selective activity via PRMT5 inhibition, we utilised the connectivity map^8^ to identify novel transcriptional suppressors of PRMT5 (Supplementary Table 3, Fig. 3A). Among the top 5 predicted molecules, Quinacrine Hydrochloride led to significant suppression of both PRMT5 mRNA levels (Fig. 3B) and protein with consequent reduction of H4R3me2S and growth arrest in MTAP negative but not MTAP positive cells (Fig. 3C). Quinacrine Hydrochloride directly inhibited PRMT5 promoter activity as confirmed by luciferase reporter assay (Fig. 3D), but did not have any direct effect on PRMT5 enzymatic activity (Fig. 3E).

Neither apoptosis nor cell cycle perturbation were observed after treatment with Quinacrine by flow cytometry, in common with both PRMT RNA interference or small molecule inhibitor (Supplementary Fig. 2A). We used a functional genetic approach to establish the extent to which the phenotypic effects of Quinacrine in MTAP negative cells were reliant upon PRMT5. Overexpression of wild-type PRMT5 rescued cells from treatment with Quinacrine, however this was not observed with transfection of the methyltransferase dead mutant PRMT5^E444Q^ compared to empty vector control. This is consistent with modulation of endogenous PRMT5 transcription as an essential mechanism underpinning the effect of Quinacrine in MTAP negative context (Fig. 3F).

### Quinacrine transcriptionally regulates PRMT5 via c-JUN

**Figure 4:**
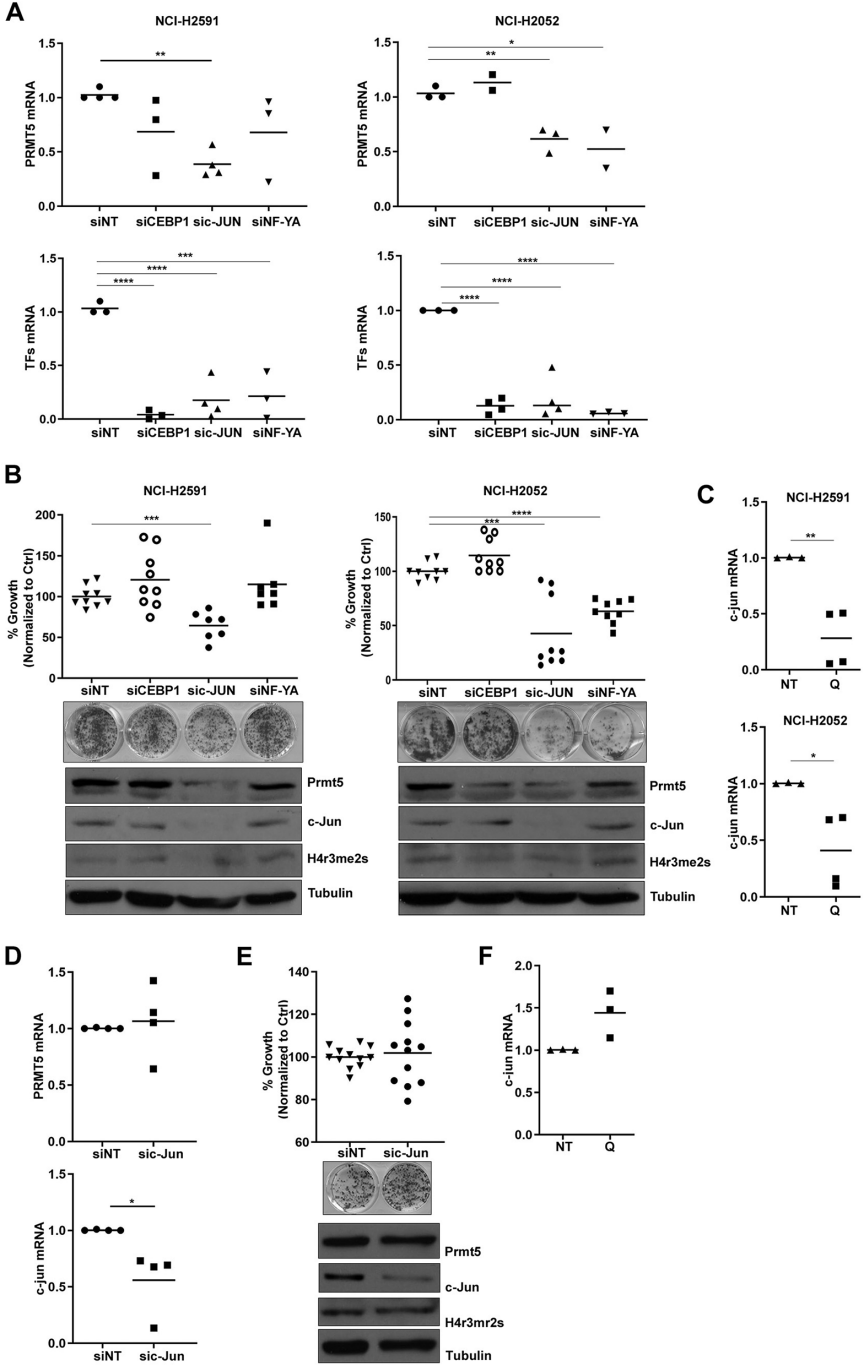
Quinacrine transcriptionally regulates PRMT5 via c-JUN (**A**) PRMT5 mRNA expression was evaluated by qRT-PCR on RNA extracted from cells transfected with siNT, siCEBP1, sic-JUN, siNF-YA 20 nM, for 72 h. Data were normalized to siNT (siCEBP1 *p* = n.s.; sic-JUN *p* = 0.0001; siNF-YA *p* = n.s). CEBP1, c-JUN and NF-YA mRNA expression was evaluated by qRT-PCR on RNA extracted from cells transfected with siNT, siCEBP1, sic-JUN, siNF-YA 20 nM, for 72 h. Data were normalized to siNT (NCI-H2591: siCEBP1 *p* = 0.0001; sic-JUN *p* = 0.0006; siNF-YA *p* = 0.0033. NCI-H2052: siCEBP1 *p* = 0.0001; sic-JUN *p* = 0.0009; siNF-YA *p* = 0.0001). (**B**) Cell proliferation was measured by clonogenic assay 5–7 days after treatment. Data were normalized to siNT (NCI-H2591: siCEBP1 *p* = n.s.; sic-JUN *p* = 0.0073; siNF-YA *p* = n.s. NCI-H2052: siCEBP1 *p* = n.s.; sic-JUN *p* = 0.0001; siNF-YA *p* = 0.0001). The levels of PRMT5 and c-JUN expression and H4 arginine 3 symmetrical di-methylation (H4R3me2S) were measured by western blot. These gels have been cropped and full length gels are presented in Supplementary Fig. 3J–K. **(C**) C-JUN mRNA expression was evaluated by qRT-PCR on RNA extracted from cells treated for 72 h with Quinacrine 1 µM. Data were normalized to untreated controls (NCI-H2591 *p* = 0.0001. NCI-H2052 *p* = 0.0276). (**D**) PRMT5 and cJUN mRNA expression was evaluated by qRT-PCR on RNA extracted from MPP89 cells transfected with siNT, sic-JUN 20 nM, for 72 h. Data were normalized to siNT (PRMT5 *p* = n.s. c-JUN *p* = 0.0205). (**E**) Cell proliferation was measured by clonogenic assay 5–7 days after treatment. Data were normalized to siNT (sic-JUN *p* = n.s). The levels of PRMT5 and c-JUN expression and H4 arginine 3 symmetrical di-methylation (H4R3me2S) were measured by western blot. This gel has been cropped and the full length gel is presented in Supplementary Fig. 3L. (**F**) C-JUN mRNA expression was evaluated by qRT-PCR on RNA extracted from cells treated for 72 h with quinacrine 1 µM. Data were normalized to untreated control *p* = n.s.

To further explore the possible mechanism of PRMT5 transcriptional perturbation by Quinacrine, we used RNA interference to screen for putative transcription factors (TFs) implicated by PROMO^9^. These TFs included CEBP1, c-JUN and NF-YA were predicted to bind to the PRMT5 promoter. RNAi mediated silencing of c-JUN, but not of CEBP1 or NF-YA, resulted in a significant reduction of PRMT5 mRNA levels in MTAP negative cells and this was comparable to that achieved with Quinacrine (Fig. 4A). c-JUN silencing led to reduced clonogenic growth with concurrent downregulation of both PRMT5 and H4R3me2S in MTAP negative cells (Fig. 4B), but no apoptosis or cell cycle perturbation (Supplementary Fig. 2B). Quinacrine suppressed c-JUN mRNA suggesting that it targets PRMT5 transcription indirectly via this transcription factor (Fig. 4C). The effects of c-JUN silencing were MTAP-selective, as MTAP positive cells failed to reduce PRMT5 mRNA (Fig. 4D), without evidence of impaired clonogenic growth or reduced H4R3me2S (Fig. 4E). Interestingly, Quinacrine did not suppress c-JUN mRNA in MTAP positive cells (Fig. 4F).

## Discussion

Copy number loss of MTAP is one of the most frequent events in MPM. Our data confirmed a marked negative prognostic effect, which warrants novel targeted therapy. It should be noted that given the co-deletion of MTAP with CDKN2A, which has been also shown to be negatively prognostic, it was not possible to deconvolute the impact of MTAP in isolation from our data involving chromosome 9p21.3 deleted cel lines. PRMT5 plays a key role in the regulation of several pathways including DNA damage response, apoptosis, inhibition of tumour suppressors, and activation of survival pathways^10^ and has been reported to be a dependency in MTAP negative cells^11^, which we have verified in MPM. We confirmed global epigenetic modification associated with reduced H4R3me2S and re-expression of tumour suppressors, such as EIF3F, FOXP4, ZBTB4, GANAB, TMEM141, in association with loss of clonogenicity.

SAM competitive PRMT5 inhibitors, such as EPZ015666 and GSK3326595, have shown limitations in recapitulating the vulnerability of MTAP negative cells in response to PRMT5 inhibition^10^. To address this limitation, we used the connectivity map approach^8^ to screen for small molecules with PRMT5 downregulating activity and identified the off-patent small molecule Quinacrine, which was primarily used as an antimalarial drug as well as an intrapleural sclerosing treatment for malignant pleural effusions with an excellent safety profile^12^. Quinacrine-mediated growth arrest was PRMT5 dependent as confirmed by a methyltransferase dead PRMT5 mutant.

Apoptosis was not observed as a mechanism of reduced clonogenicity; the phenotype was cytostatic, but not restricted to any phase of the cell cycle. Recent studies suggest that small molecule PRMT5 inhibition, either directly or indirectly through inhibition of cyclin dependent kinases 4 and 6, alters RNA splicing, leading to an MDM4 dependent activation of p53/p21^13,14^. Whether this axis is exploited by Quinacrine requires further exploration.

Quinacrine has been reported to regulate c-jun phosphorylation at positions 349 to 340 and 266 to 257^15^. We showed that c-jun is essential in driving PRMT5 expression and mediating Quinacrine-induced apoptosis. This study therefore implicates c-jun or SAPK pathway regulation as a mechanism underlying PRMT5 transcriptional repression by Quinacrine.

Based on early pharmacokinetic studies conducted with Quinacrine in the 1940′s, plasma concentration was measured as 225 nM at a dose of 100 mg, 3 times a day. However, hepatic and leucocyte distributions are high, with high plasma binding. This agent can however be safely instilled into the pleural cavity at a much higher dose of 5 mM, at which dose it acts as a sclerosant. This implies that lower micromole doses are achievable and could be locally administered, achieving localised exposure to mesothelioma at concentrations capable of suppressing PRMT5 expression.

Our results provide a proof of concept to support the use of small molecule transcriptional perturbation to leverage a somatic-mutation based vulnerability, suggesting a repurposing potential that warrants further study.

## Material and methods

### Patient samples

Seventy-nine patient MPM samples were obtained at the time of extended pleurectomy decortication (EPD) under ethical approval. This study was approved by the London-Fulham Research Ethics Committee (reference 14/LO/1527), The Northampton Research Ethics Committee (reference 14/EM/1159) and University Hospitals Leicester NHS Trust Research and Innovation Department (reference UHL 11,363). Patient clinico-pathological characteristics are outlined in Supplementary Table I. Overall survival was calculate from the date of surgery. A separate cohort of 100 patients^16^ was used for validation and clinico-pathological characteristics are described in Supplementary Table I. Informed consent to provide research samples was obtained from all patients. All methods were carried out in accordance with local guidelines and regulations.

### Oncoscan analysis

DNA was extracted with the GeneRead DNA FFPE kit (Qiagen, Manchester, UK). 80 ng of gDNA were analysed using the OncoScan FFPE Assay Kit (Affymetrix, Wooburn Green High Wycombe, UK), which utilizes molecular inversion probe (MIPs) technology^17^. The BioDiscovery Nexus Express 10.0 for OncoScan software was then used to define copy number alterations and loss of heterozygosity as previously described^18^.

### Reagents and antibodies

Quinacrine was purchased from Sigma (Gillingham, UK). The antibodies against MEP50, PRMT5 and c-JUN were obtained from Cell Signaling (Hitchin, UK), MTAP antibody was purchased from Santacruz (Wembley, UK), H4R3me2S and β-tubulin were obtained from Abcam (Cambridge, UK). Goat anti-rabbit HRP and donkey anti-mouse HRP secondary antibodies were from Cell Signaling (Hitchin, UK).

### Cell lines

MPM cell lines: NCI-H2052, were purchased from ATCC (Middlesex, UK). MPP89 and NCI-H2591 were kindly provided by Dr. P.W. Szlosarek, Institute of Cancer at Barts, London, UK. Cell lines were grown in RPMI Medium 1640, 1% L-Glutamine and 10% FBS (Gibco, Loughborough, UK).

### Clonogenic assays

5000 cells per well were seeded in 12 well plates and left untreated or treated with Quinacrine (500 nM, 1 µM, 2 µM). Cells were fixed between days five and seven (once enough colonies had formed in the control) on ice in methanol for 10 min. Cells were then stained with crystal violet (Sigma, Gillingham, UK) for 20 min. Colonies were dissolved in 30% acetic acid to allow quantification^19^. Each treatment condition was measured in triplicate.

### siRNA transfections

The non-silencing control (NT), siPRMT5, siWDR77, sic-JUN, siCEBPB, siNF-YA were obtained from Qiagen (Manchester, UK). siRNA transfections (20 nM) were performed using the RNAiMAX transfection reagent (Invitrogen, Paisley, UK) according to manufacturer’s instructions. Validation of siPRMT5 and siWDR77 was carried out with a second sequence from Dharmacon (Little Chalfont, UK).

### Real time proliferation assay

The xCELLigence RTCA DP instrument (Acea Bioscience, San Diego, CA) was used as described in the manufacturer´s instruction manual. Cells (5,000 cells/ well) were seeded in E16-Plates. The cell indices were measured every 15 min for 120 h. Each treatment condition was measured in triplicate.

### Connectivity mapping

A PRMT5-centred gene signature was created from co-expression analyses of 9 independent Gene Expression Omnibus (GEO) datasets GSE37745, GSE50081, GSE28571, GSE77803, GSE43580, GSE19804, GSE18842, GSE10245, and GSE19188. Within each dataset, PRMT5 was used as the seed gene, with which the gene expression correlation coefficients for other genes (probes) were calculated. All genes were then ranked based on the magnitude and statistical significance of their correlations with the seed, following the ranking method described in^18^. The genes’ ranks were then combined across these datasets to obtain an overall rank for each gene to determine its inclusion to the PRMT5 gene signature for subsequent connectivity mapping analysis. A gene signature progression approach^19^ determined that an 8-gene signature was the optimal length including PRMT5 and its 7 strongest co-expression correlates PSMB5, HNRNPC, APEX1, HNRNPC, IPO4, TOX4, and TUBB. This 8-gene signature was used as an input to query a collection of 83,939 reference drug gene expression profiles^20^ covering 1353 FDA approved drugs (http://www.lincscloud.org). This connectivity mapping analysis was conducted in the framework of sscMap^21,22^. In our analysis all the individual reference profiles with the same drug formed a reference set. A set score was then calculated between the gene signature and each reference set, and the associated *p*-value was estimated by generating a large number of random gene signatures of the same length. Any signature-drug connections with a *p*-value no greater than a pre-set threshold (1/1353 = 7.4e−4) were declared as statistically significant. Additionally, gene signature perturbation analysis^23^ was performed to obtain the robustness (perturbation stability) of the significant signature-drug connections. Only the significant drugs that had 100% perturbation stability were selected for further consideration. Finally, significant drugs were ranked by the absolute value of their connection z-score to the PRMT5 gene signature.

### PRMT5 enzymatic activity

PRMT5 chemoluminescent assay was purchased from AMS Biotechnology (Europe) Ltd (Abingdon, UK). Quinacrine or EPZ015666 was added to the plate pre-coated with histone H4 peptide substrate. PRMT5 enzymatic activity was measured after reaction with the antibody against methylated arginine3 residue of Histone H4, the secondary HRP-labeled antibody, S-adenosylmethionine, methyltransferase assay buffer, and purified PRMT5 enzyme, according to the manufacturer instructions.

### Protein extraction and immunoblotting

Seventy-two hours after treatment cells were lysed in RIPA buffer containing protease inhibitors (Roche, Burgess Hill, UK). Lysates were clarified by centrifugation at 4 °C at 13,000 rpm for 10 min. 40 µg of total cell lysates were loaded on SDS-PAGE gels. Signal detection was performed with ECL-plus chemiluminescent system (GE Healthcare, Little Chalfont, UK).

### Real time quantitative RT-PCR

Total RNA was extracted using Trizol (Invitrogen, Paisley, UK) according to manufacturer’s instructions. Reverse transcription was performed with High Capacity RNA-to-cDNA Kit (Applied Biosystem, Paisley, UK). Real-Time PCR was carried out using Power SYBR Green PCR Master Mix (Applied Biosystem, Paisley, UK) after 72 h of silencing or treatment with Quinacrine. QuantiTect primer assays (Qiagen, Manchester, UK) were used for PRMT5, c-JUN and Actin.

### Reporter assay

Cells were transfected with pGL2 basic or pGL2-PRMT5 and Renilla by using the Xtreme gene transfection reagent (Sigma, Gillingam, UK) according to the manufacturer’s instructions. After 24 h cells were treated with Quinacrine and 72 h after transfection cells were lysed and stored at -80 °C for at least 24 h**.** The luciferase activity was then measured by a Dual-Luciferase reporter assay system (Promega, Southampton, UK). Luciferase activity was normalized to Renilla activity^19^.

### Overexpression of PRMT5

NCI-H2591 cells were transfected with 2 µg of PRMT5 (WT or mutant) or empty vector, using the Xtreme gene transfection reagent (Sigma, Gillingham, UK) according to the manufacturer’s instructions. Selection of stable clones was performed with G418 500 μg/ml (Sigma, Gillingham, UK).

### Site directed mutagenesis

The Quick change II site directed mutagenesis kit (Agilent, Cheadle, UK) was used according to manufacturer instructions to introduce a mutation in PRMT5 (E444Q).

### Statistical analysis

Dose–response curves were fitted using non-linear regression (GraphPad Prism version 6.0, GraphPad Software, LaJolla, CA, USA). The significance of the data has been assessed with *t*-test (two tails), or One-way ANOVA. All data are representative of the mean and standard deviation for at least three independent experiments. Kaplan–Meier curves for overall survival were used to assess survival estimates of the cohorts. Univariate analyses comparing the clinical variables with overall survival were performed using the log rank test and all statistically significant clinical variables were taken forward into a multivariate Cox regression model. The data was analysed using SPSS Version 25 (Armonk, NY, IBM Corp). All *p*-values less than or equal to 0.05 were considered significant.

## Supplementary Information

Supplementary Information
