# Supplementary material for: Transcriptional perturbation of protein arginine methyltransferase-5 exhibits MTAP-selective oncosuppression
Source: Sci Rep. 2021 Apr 1;11:7434. doi: 10.1038/s41598-021-86834-7 (PMC8016828; doi:10.1038/s41598-021-86834-7)
Supplement: Supplementary file 1 — Supplementary Information [file 41598_2021_86834_MOESM1_ESM.pdf]

Transcriptional Perturbation of Protein Arginine Methyltransferase-5 exhibits MTAP-selective oncosuppression

Sara Busacca[1], Qi Zhang[2], Annabel Sharkey[1], Alan G. Dawson[1,6], David A. Moore[3,4], David A. Waller[5], Apostolos Nakas[6], Carolyn Jones[7], Kelvin Cain[7], Jin-li Luo[1], Adriana Salcedo[8,9,10], Iris Salaroglio[11], Chiara Riganti[11], John Le Quesne[1,7], Tom John[12], Paul C. Boutros[8], Shu-Dong Zhang[13] and Dean A. Fennell[1]*

SUPPLEMENTARY TABLES

Supplementary table I. Patient characteristics

	UK cohort (n=79)		AUS cohort (n=100)	
	**MTAP CN loss (n=33)**	**MTAP WT (n=46)**	**MTAP CN loss (n=63)**	**MTAP WT (n=37)**
**Age at diagnosis (median (IQR))**	66 (61-74)	63 (55-70)	69 (62-74)	64 (57-74)
**Sex (N (%))**				
**M**	29 (88)	38 (83)	53 (84)	28 (76)
**F**	4 (12)	8 (17)	10 (16)	9 (24)
**Survival status (N (%))**				
**Alive**	1 (3)	0 (0)	4 (6)	6 (16)
**Dead**	32 (97)	46 (100)	59 (94)	31 (84)

**Overall survival (months) (median (95% CI)**	12.5 (95%CI: 6.8-18.2)	17.6 (95%CI: 6.5-28.7)	8.7 (95%CI: 4.6-12.8)	22.7 (95% CI: 11.4-33.9)
**IMIG Stage (N (%))**				
**1**	0 (0)	1 (2)	2 (3)	3 (8)
**2**	4 (12)	7 (15)	24 (38)	14 (38)
**3**	24 (73)	28 (61)	30 (48)	17 (46)
**4**	5 (15)	10 (22)	7 (11)	3 (8)
**Histology (N (%))**				
**Epithelioid**	28 (85)	43 (93)	37 (59)	26 (70)
**Biphasic**	5 (15)	3 (7)	18 (28)	4 (11)
**Sarcomatoid**	0 (0)	0 (0)	8 (13)	7 (19)
**Neoadjuvant chemotherapy administered (N (%))**	1 (3)	4 (9)	1 (2)	0 (0)

Supplementary table 2 GSEA analysis

Entrez Gene Id	Gene Symbol	LU EZH2 TARGETS UP	GO ENZYME BINDING	GO PHOSPHATASE INHIBITOR ACTIVITY	GGGCGGR SP1 06	GO RAB GTPASE BINDING	GGGAGGRR MAZ 06	GO GTPASE BINDING	GARY CD5 TARGETS UP	GO PHOSPHATASE REGULATOR ACTIVITY	WAKABAYASHI ADIPOGENESIS PPARG RXRA BOUND 8D	GSE13306 TREG VS TCONV UP	GSE14308 TH1 VS TH17 DN	GSE14308 TH2 VS TH17 DN	GSE17721 CTRL VS PAM3CSK4 8H BMDC UP	GSE17721 LPS VS POLYIC 4H BMDC DN	GSE21063 3H VS 16H ANTI IGM STIM BCELL DN	GSE22611 MUTANT NOD2 TRANSDUCED VS CTRL HEK293T STIMULATED WITH MDP 6H DN	GSE41176 UNSTIM VS ANTI IGM STIM TAK1 KO BCELL 24H UP	GSE45837 WT VS GFI1 KO PDC DN	CCAGGGG MIR331	Entrez	Source	Gene Description
2029	ENSA	■	■	■	■		■			■										■	■		S	endosulfine alpha
114794	ELFN2	■	■	■						■													S	extracellular leucine-rich repeat and fibronectin type III domain containing 2
26472	PPP1R14B	■		■					■	■						■							S	protein phosphatase 1, regulatory (inhibitor) subunit 14B
23193	GANAB	■			■						■									■			S	glucosidase, alpha; neutral AB
22927	HABP4	■			■								■		■			■					S	hyaluronan binding protein 4
85014	TMEM141	■			■											■							S	transmembrane protein 141

11054	OGFR	■							■									■	■				S	opioid growth factor receptor
84717	HDGFRP 2	■									■	■											S	hepatoma-derived growth factor-related protein 2
84263	HSDL2	■									■		■	■			■						S	hydroxysteroid dehydrogenase like 2
85364	ZCCHC3	■													■								S	zinc finger, CCHC domain containing 3
56993	TOMM22	■													■								S	translocase of outer mitochondrial membrane 22 homolog (yeast)
11487 9	OSBPL5	■																					S	oxysterol binding protein-like 5
84331	FAM195 A	■																					S	family with sequence similarity 195, member A
90317	ZNF616	■																					S	zinc finger protein 616
84687	PPP1R9B		■	■			■		■	■	■												S	protein phosphatase 1, regulatory subunit 9B
11570 4	EVI5L		■		■	■	■	■			■	■											S	ecotropic viral integration site 5-like
2801	GOLGA2		■		■		■											■					S	golgin A2
57659	ZBTB4		■		■		■																S	zinc finger and BTB domain containing 4
5532	PPP3CB		■		■												■						S	protein phosphatase 3, catalytic subunit, beta isozyme
23258	DENND5 A		■			■	■	■															S	DENN/MADD domain containing 5A
26000	TBC1D10 B		■			■		■													■		S	TBC1 domain family, member 10B
23061	TBC1D9 B		■			■		■															S	TBC1 domain family, member 9B (with GRAM domain)
23299	BICD2		■			■		■															S	bicaudal D homolog 2 (Drosophila)
4720	NDUFS2		■				■							■	■			■	■				S	NADH dehydrogenase (ubiquinone) Fe-S protein 2, 49kDa

			■				■							■	■			■	■					(NADH-coenzyme Q reductase)
10811	NOXA1		■					■															S	NADPH oxidase activator 1
5886	RAD23A		■																				S	RAD23 homolog A (S. cerevisiae)
59	ACTA2		■																				S	actin, alpha 2, smooth muscle, aorta
11193	WBP4				■		■						■										S	WW domain binding protein 4 (formin binding protein 21)
29924	EPN1				■		■																S	epsin 1
57804	POLD4				■				■		■			■					■				S	polymerase (DNA-directed), delta 4
115209	OMA1				■											■							S	OMA1 homolog, zinc metallopeptidase (S. cerevisiae)
2802	GOLGA3				■												■						S	golgin A3
374920	C19orf68				■																		S	chromosome 19 open reading frame 68
5433	POLR2D				■																		S	polymerase (RNA) II (DNA directed) polypeptide D
116113	FOXP4				■																		S	forkhead box P4
51754	TMEM8B				■																		S	transmembrane protein 8B
11093	ADAMTS13				■																		S	ADAM metallopeptidase with thrombospondin type 1 motif, 13
29123	ANKRD11						■		■					■									S	ankyrin repeat domain 11
22864	R3HDM2						■		■														S	R3H domain containing 2
2036	EPB41L1						■					■											S	erythrocyte membrane protein band 4.1-like 1
84265	POLR3GL						■																S	polymerase (RNA) III (DNA directed) polypeptide G (32kD)-like
7461	CLIP2						■																S	CAP-GLY domain containing linker protein 2
23373	CRTC1						■																S	CREB regulated transcription coactivator 1
25875	LETMD1								■		■												S	LETM1 domain containing 1

79144	PPDPF										■	■											S	pancreatic progenitor cell differentiation and proliferation factor homolog (zebrafish)
54760	PCSK4										■												S	proprotein convertase subtilisin/kexin type 4
9790	BMS1											■											S	BMS1 homolog, ribosome assembly protein (yeast)
8665	EIF3F												■							■			S	eukaryotic translation initiation factor 3, subunit F
7586	ZKSCAN1												■										S	zinc finger with KRAB and SCAN domains 1
1632	ECI1													■		■				■			S	enoyl-CoA delta isomerase 1
166	AES														■								S	amino-terminal enhancer of split
23265	EXOC7															■	■				■		S	exocyst complex component 7
55624	POMGNT1																■	■					S	protein O-linked mannose beta1,2-N-acetylglucosaminyltransferase
3913	LAMB2																		■				S	laminin, beta 2 (laminin S)
8733	GPAA1																		■				S	glycosylphosphatidylinositol anchor attachment protein 1 homolog (yeast)
203069	R3HCC1																			■			S	R3H domain and coiled-coil containing 1
23351	KHNYN																				■		S	KH and NYN domain containing
23229	ARHGEF9																						S	Cdc42 guanine nucleotide exchange factor (GEF) 9
60343	FAM3A																						S	family with sequence similarity 3, member A
25870	SUMF2																						S	sulfatase modifying factor 2
593	BCKDHA																						S	branched chain keto acid dehydrogenase E1, alpha polypeptide

144348	ZNF664																						S	zinc finger protein 664
79090	TRAPPC6A																						S	trafficking protein particle complex 6A
80854	SETD7																						S	SET domain containing (lysine methyltransferase) 7
642852	LOC642852																						S	uncharacterized LOC642852
57647	DHX37																						S	DEAH (Asp-Glu-Ala-His) box polypeptide 37
7637	ZNF84																						S	zinc finger protein 84
113675	SDSL																						S	serine dehydratase-like
60686	C14orf93																						S	chromosome 14 open reading frame 93
23408	SIRT5																						S	sirtuin 5
554203	JPX																						S	JPX transcript, XIST activator (non-protein coding)
90321	ZNF766																						S	zinc finger protein 766
93621	MRFAP1																						S	Morf4 family associated protein 1
79088	ZNF426																						S	zinc finger protein 426
100131564	LOC100131564																						S	uncharacterized LOC100131564

Supplementary Table 3 Connectivity map analysis

Drug	Replicate	p value	Z score
BRD-K71103788__duloxetine hcl	48	1.85E-10	-6.2659907
BRD-K75641298__METOCLOPRAMIDE HYDROCHLORIDE	29	2.77E-09	-5.8298123
BRD-K36862742__hydro-flumethiazide	141	3.04E-08	-5.4165957
BRD-K99964838__S1014	66	6.16E-08	-5.2888016
BRD-A45889380__QUINACRINE HYDROCHLORIDE	206	8.03E-08	-5.240004
BRD-K91699951__benzonatate	234	1.39E-07	-5.1383754
BRD-K71879491__ATRA	205	2.17E-07	-5.0531872
BRD-A51820102__econazole	36	2.22E-07	-5.0489293
BRD-K37270826__mifepristone	125	2.62E-07	-5.0175175
BRD-K70487031__cis-(Z)-FLUPENTHIXOL	27	3.03E-07	-4.9893544
BRD-K63675182__triflupromazine	179	5.12E-07	-4.8868451
BRD-K89375097__pirenzepine	36	6.96E-07	-4.826062
BRD-K19706299__MRE-269	99	7.54E-07	-4.8102311
BRD-K32164935__TOLAZAMIDE	353	1.17E-06	-4.7219067
BRD-K28936863__ketotifen	85	1.18E-06	-4.7204909
BRD-A23723433__paclitaxel	48	1.22E-06	-4.7138292
BRD-K39621635__artemether	34	1.25E-06	-4.7081455
BRD-K81418486__SAHA__trt_poscon	12	1.36E-06	-4.6910799
BRD-K92093830__doxorubicin	159	1.58E-06	-4.6599437
BRD-A49160188__donepezil	73	1.74E-06	-4.6404777
BRD-K70487031__flupentixol	37	2.14E-06	-4.5971052
BRD-K10916986__S1527	67	2.20E-06	-4.5915996
BRD-K92778217__mefenamic-acid	160	2.39E-06	-4.5741376
BRD-K13514097__S1120	58	3.79E-06	-4.4764562
BRD-K45995181__Auranofin	3	3.84E-06	-4.474083
BRD-K66175015__S1011	59	4.68E-06	-4.4316279
BRD-K53737926__amitryptiline	137	4.95E-06	-4.4194573
BRD-K52075040__cerulenin	210	5.16E-06	-4.4103748
BRD-K20075662__betazole	143	5.27E-06	-4.4059091
BRD-K33106058__cytarabine	48	5.52E-06	-4.395691
BRD-K91290917__Amodiaquin dihydrochloride dihydrate	57	5.65E-06	-4.3905396
BRD-K81418486__vorinostat	1303	6.50E-06	-4.360225
BRD-K93754473__tamoxifen	843	6.59E-06	-4.3571007
BRD-K25433859__maprotiline	37	9.22E-06	-4.2829289
BRD-K39339537__epirizole	217	1.16E-05	-4.2316941
BRD-A45889380__Quinacrine dihydrochloride dihydrate	10	1.58E-05	-4.1619013
BRD-K81528515__HY-10159	66	1.60E-05	-4.1585908

BRD-K17016787__estriol	355	1.78E-05	-4.1344654
BRD-K21680192__mitoxantrone	334	1.91E-05	-4.1181513
BRD-K41260949__Sodium Valproate	45	2.33E-05	-4.0721889
BRD-K39987650__BISACODYL	93	2.54E-05	-4.0520213
BRD-K89997465__CHLORPROMAZINE	86	2.68E-05	-4.0390003
BRD-K81418486__VORINOSTAT__trt_poscon	2575	3.07E-05	-4.0072094
BRD-K63750851__mycophenolic-acid	31	3.78E-05	-3.9582377
BRD-K47869605__podofilox	72	3.85E-05	-3.9538222
BRD-K61250553__Loperamide.HCl	23	3.91E-05	-3.9501318
BRD-K20168442__vecuronium	49	4.36E-05	-3.9234926
BRD-K61250553__imodium	45	4.38E-05	-3.9224636
BRD-A29734509__norpace	44	5.30E-05	-3.8764725
BRD-K28143534__Cyproheptadine hydrochloride	10	1.07E-04	-3.7019795
BRD-K86434416__deprenalin	88	1.31E-04	-3.6498136

SUPPLEMENTARY METHODS

Clonogenic assays

5000 cells per well were seeded in 12 well plates and left untreated or treated with EPZ015666 (1 µM, 5 µM, 10 µM). The PRMT5 inhibitor EPZ015666 was obtained from Selleckchem (Ely, UK).

Five days after treatment, cells were fixed on ice in methanol for 20 minutes. Cells were then stained with crystal violet (Sigma, Gillingham, UK) for 10 minutes. Colonies were dissolved in 30% acetic acid to allow quantification. Each treatment condition was measured in triplicate.

siRNA transfections

The non-silencing control (NT) and siRIOK, were obtained from and Qiagen (Manchester, UK). siRNA transfections (20 nM) were performed using the RNAiMAX transfection reagent (Invitrogen, Paisley, UK) according to manufacturer’s instructions.

Flow Cytometry

Samples were analysed on a BD FACS Calibur flow cytometer machine, using Cell Quest Pro software (Becton Dickinson, Oxford, UK). Cells were fixed after 16, 32, 72 and 120 hours of treatment with quinacrine, EPZ015666 or after silencing of PRMT5 and stained with propidium iodide (Sigma).

Gene expression

NCI-H2591 and NCI-H2052 cells were transfected with non-silencing control (NT), siPRMT5 Qiagen and siPRMT5 Dharmacon. RNA was extracted 120h after transfection with the RNeasy Mini Kit (Qiagen, Manchester, UK) and 100 ng of RNA were hybridised on the SurePrint G3 Human Gene Expression v3 8x60k array (Agilent, Cheadle, UK).

Gene expression dataset was analysed using Limma in R/Bioconductor software package. Comparisons were performed siQ vs siNT, siD vs siNT. Gene lists were then filtered by fold change ≥2, unadjusted p value ≤ 0.05. Webgestalt (http://www.webgestalt.org/) was used to perform Gene Ontology, KEGG pathway, Pathway Common, Wiki Pathway. GSEA Molecular Signatures Database (http://software.broadinstitute.org/gsea/msigdb/index.jsp) was used to investigate 85 upregulated gene set.

SUPPLEMENTARY FIGURE LEGENDS

Supplementary Figure 1. A) Cells were transfected with siNT or siRIOK1 20 nM. Cell proliferation was measured by clonogenic assay. Data were normalized to siNT controls (NCI-H2591: p= n.s.; NCI-H2052: p=0.0001; NCI-H2452: p=n.s; MPP89: p= n.s.). The levels of PRMT5 expression and H4 arginine 3 symmetrical di-methylation (H4R3me2S) were measured by western blot 72 hours after transfection. These gels have been cropped and the full length gels are presented in Supplementary Figure 3 M-O**. B)** Flow cytometry plot showing cells transfected with siNT or siPRMT5 for 120 hours. **C)** Cells were left untreated or treated with EPZ015666 1μM, 5 μM and 10μM. Cell proliferation was measured by clonogenic assay 5-7 days after treatment. Data were normalized to untreated controls (NCI-H2591: 1 μM p=0.0176; 5 μM p=0.0001; 10 μM p=0.0001; NCI-H2052: 1 μM p=n.s.; 5 μM p= n.s. 10 μM p=0.0033; MPP89: 1 μM p=n.s.; 5 μM p= n.s. 10 μM p= n.s.). **D)** Flow cytometry plot showing cells left untreated or treated with EPZ015666 5 μM and 10μM for 72 hours.

Supplementary Figure 2. Flow cytometry plot showing cells left untreated or treated with quinacrine 1 μM for 120 hours. **B)** Flow cytometry plot showing cells transfected with siNT, sic-JUN 20 nM, for 120 hours.

Supplementary Figure 3. Uncropped images of full length gels used throughout the manuscript. **A-C:** Full length gels of cropped gels in Figure 1C (A: NCI-H2591; B: NCI-H2052; C: MPP89); **D-F:** Full length gels of cropped gels in Figure 1D (D: NCI-H2591; E: NCI-2052; F: MPP89); **G-I:** Full length gels of cropped gels in Figure 3C (G: NCI-H2591; H: NCI-H2052; I: MPP89); **J-K:** Full length gels of cropped gels in Figure 4B (J: NCI-H2591; K: NCI-H2052); **L:** Full length gel of cropped gel in Figure 4E; **M-O:**

Full length gels of cropped gels in Supplementary Figure 1A (M: NCI-H2591; N: NCI-H2052; O: MPP89).

Supplementary Figure 1.

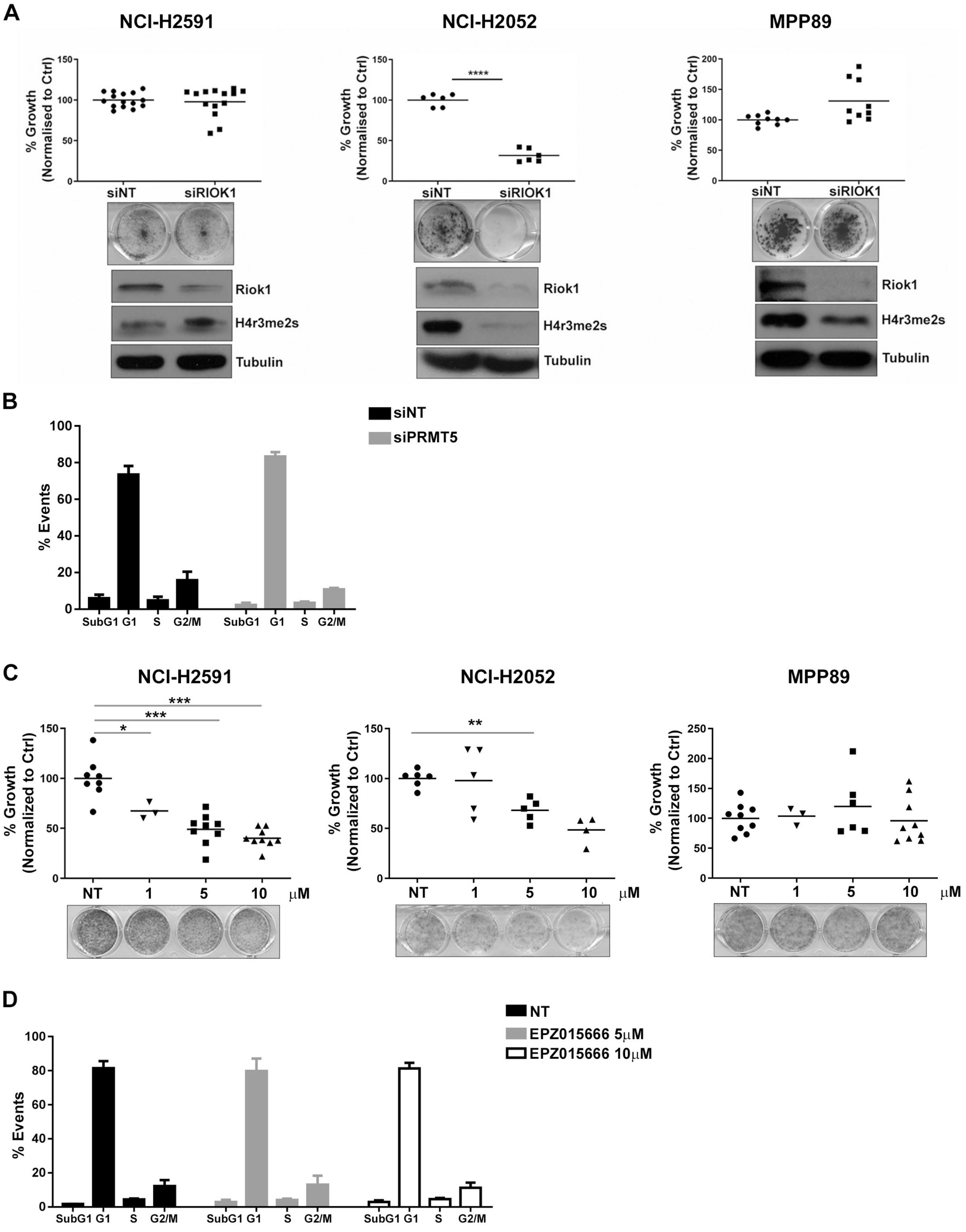

Supplementary Figure 2.

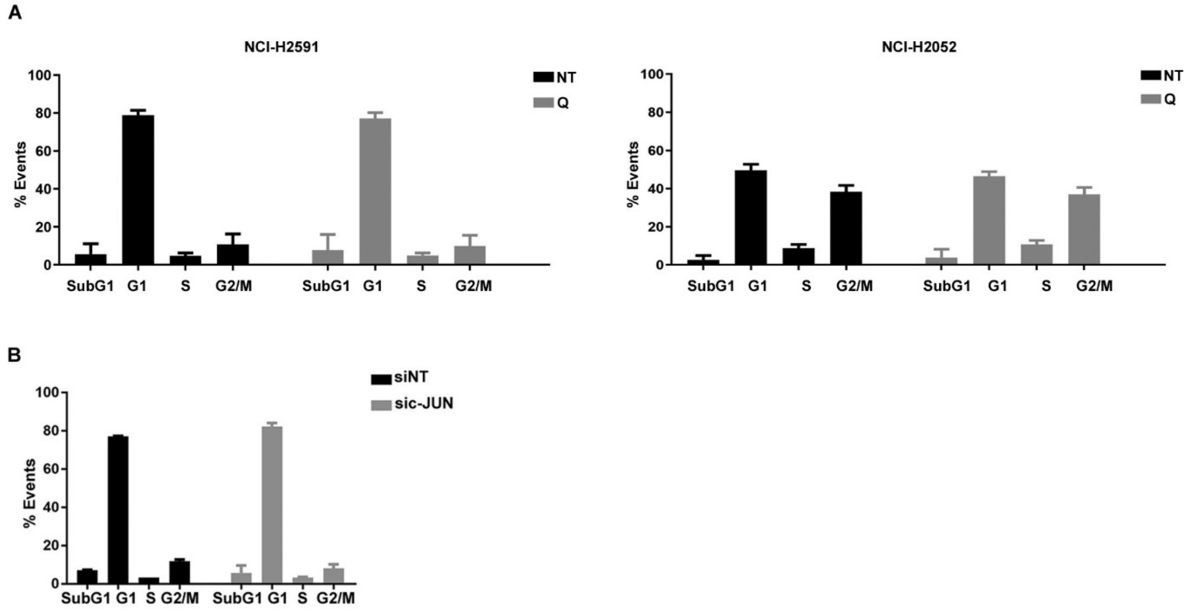

Supplementary Figure 3.

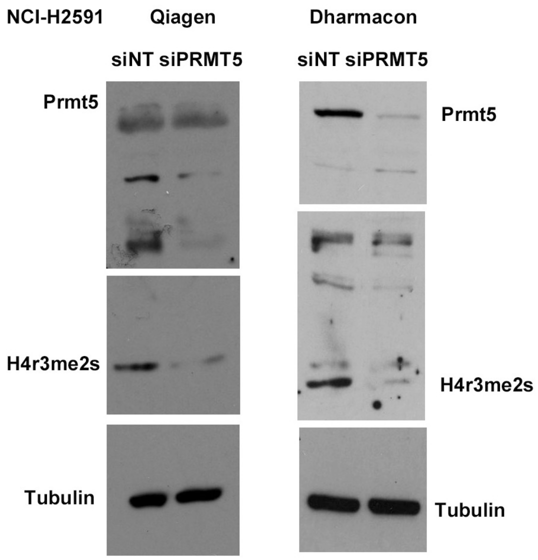

A

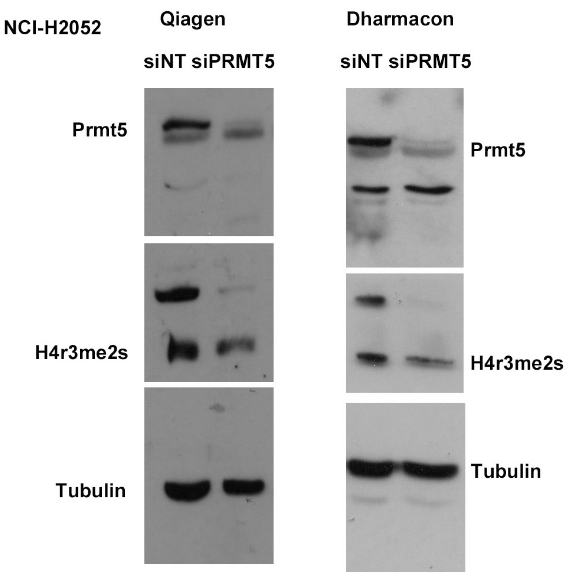

B

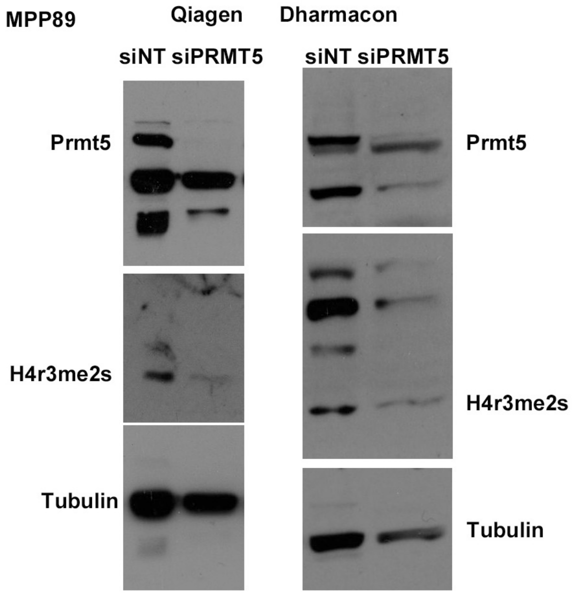

C

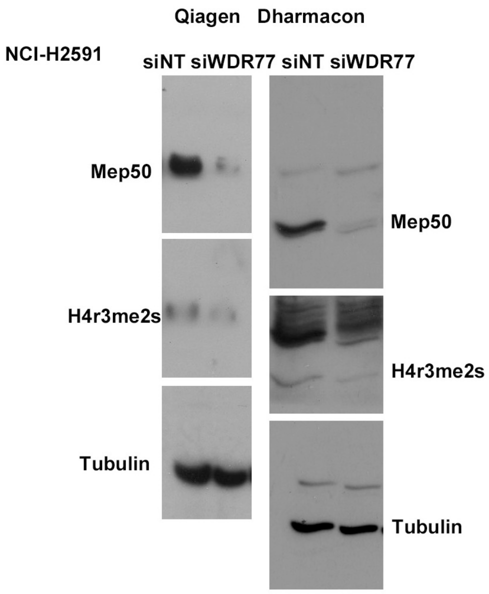

D

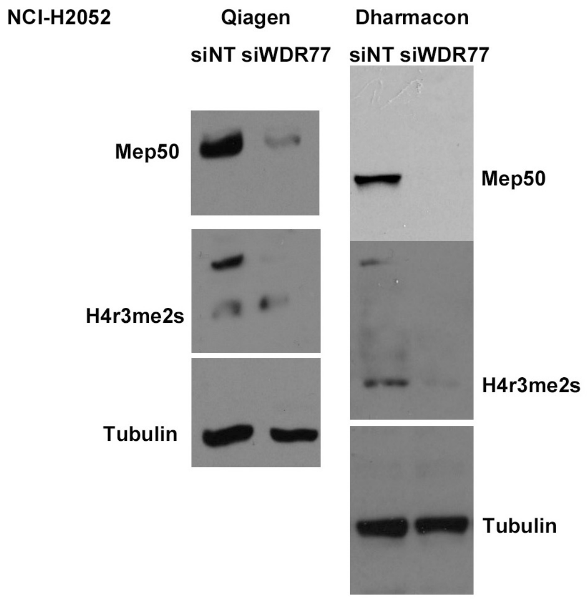
NCI-H2052
Qiagen
Dharmacon
siNT siWDR77
siNT siWDR77
Mep50
Mep50
H4r3me2s
H4r3me2s
Tubulin
Tubulin

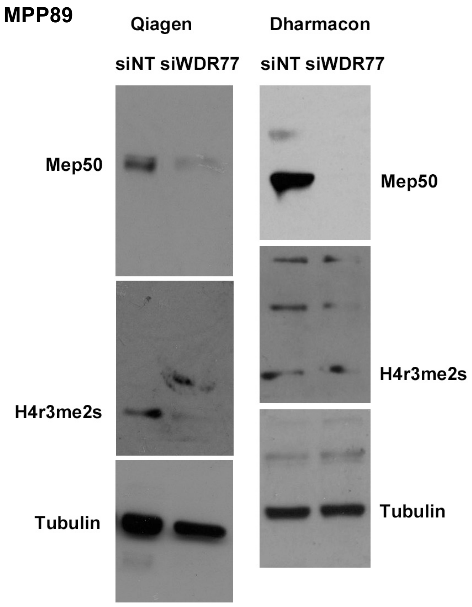
MPP89
Qiagen
Dharmacon
siNT siWDR77
siNT siWDR77
Mep50
Mep50
H4r3me2s
H4r3me2s
Tubulin
Tubulin

E

F

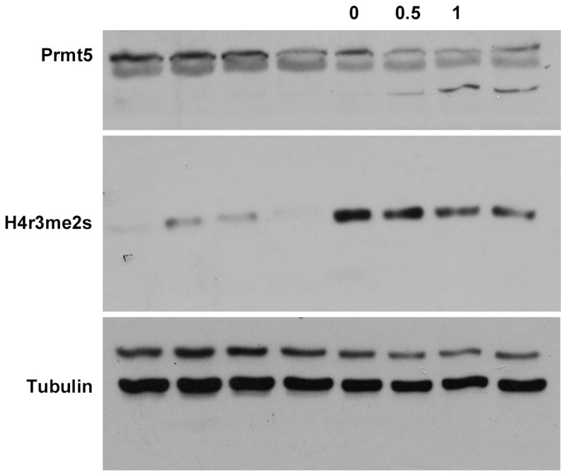
0
0.5
1
Prmt5
H4r3me2s
Tubulin

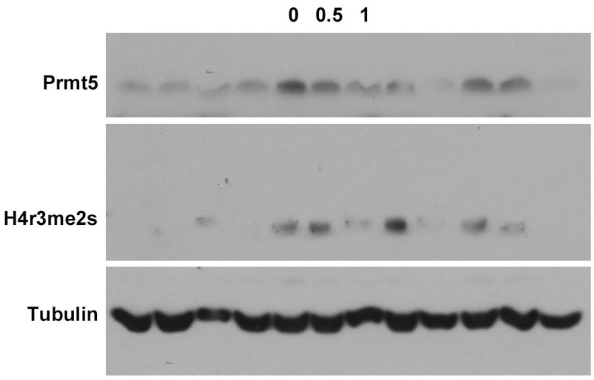
0
0.5
1
Prmt5
H4r3me2s
Tubulin

G

H

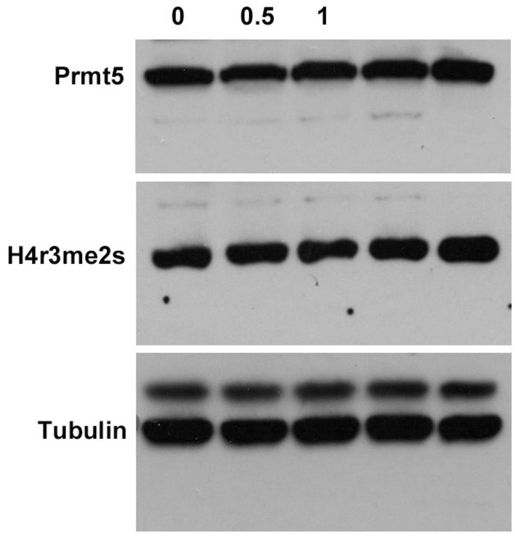
0
0.5
1
Prmt5
H4r3me2s
Tubulin

I

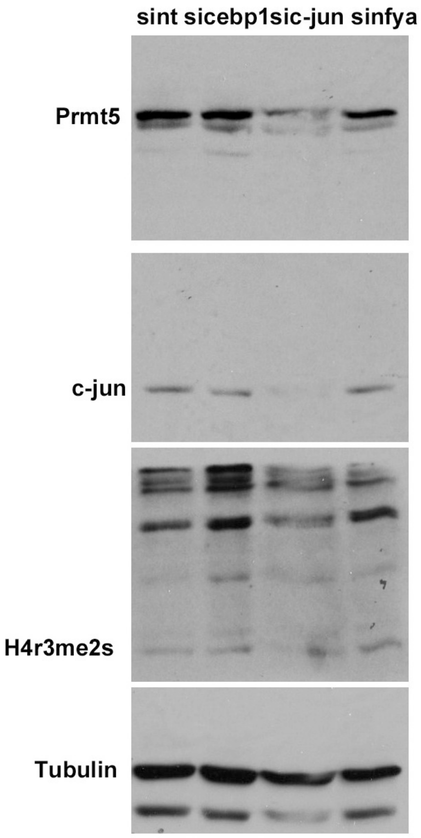
sint sicebp1sic-jun sinfya
Prmt5
c-jun
H4r3me2s
Tubulin

J

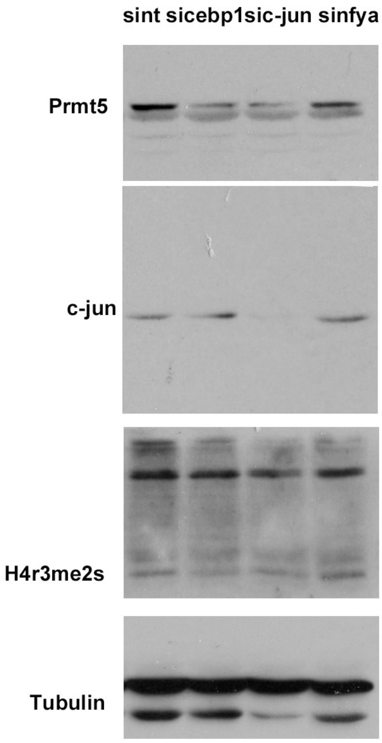
sint sicebp1sic-jun sinfya
Prmt5
c-jun
H4r3me2s
Tubulin

K

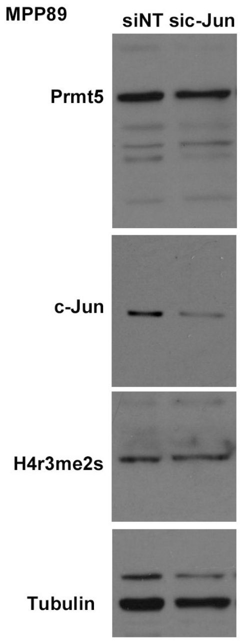
MPP89
siNT sic-Jun
Prmt5
c-Jun
H4r3me2s
Tubulin

L

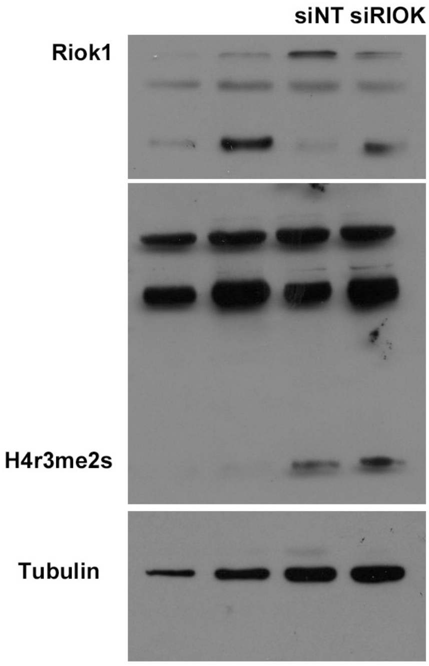
siNT siRIOK
Riok1
H4r3me2s
Tubulin

M

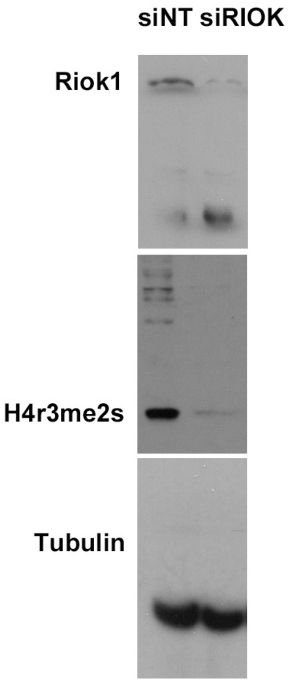
siNT siRIOK
Riok1
H4r3me2s
Tubulin

N

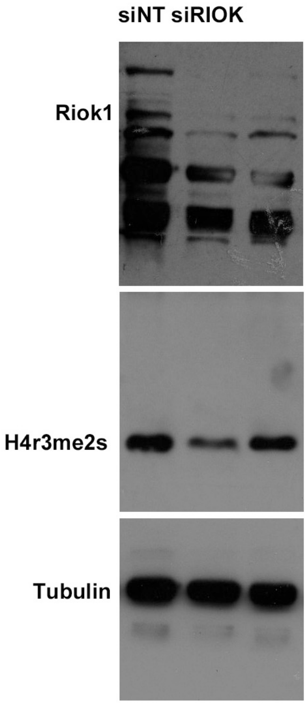
siNT siRIOK
Riok1
H4r3me2s
Tubulin

O
